# The prevalence and associated factors of depression among patients with schizophrenia in Addis Ababa, Ethiopia, cross-sectional study

**DOI:** 10.1186/s12888-019-2419-6

**Published:** 2020-01-02

**Authors:** Tolesa Fanta, Desalegn Bekele, Getinet Ayano

**Affiliations:** 1 Research and training department, Amanuel mental specialized hospital, Po Box 1971, Addis Ababa, Ethiopia; 2 Department of psychiatry, Addis Ababa University, Addis Ababa, Ethiopia

**Keywords:** Depression, Prevalence, Comorbidity, Schizophrenia, Ethiopia

## Abstract

**Background:**

Depression is common among people with schizophrenia and associated with severe positive and negative symptoms, higher rates of disability, treatment resistance and mortality related to suicide, physical and drug-related causes. However, to our knowledge, no study has been conducted to report the magnitude of depression among people with schizophrenia in Ethiopia. Therefore, this study aimed to determine the prevalence and associated factors of depression among people with schizophrenia.

**Method:**

A hospital-based cross-sectional study was conducted among 418 patients with schizophrenia selected by systematic sampling technique. Patient Health Questionnaire 9 (PHQ-9) was used to measure depression among the study participants. To identify the potential contributing factors, we performed binary and multivariable logistic regression analysis adjusting the model for the potential confounding factors. Odds ratios (OR) with the corresponding 95% confidence interval (95%CI)) was determined to evaluate the strength of association.

**Result:**

The prevalence estimate of depression among people with schizophrenia was found to be 18.0% [95% confidence interval: 14.50–22.30]. Our multivariable analysis revealed that current substance use (AOR 2.28, 95%CI (1.27, 4.09), suicide attempt (AOR 5.24, 95%CI (2.56, 10.72), duration of illness between 6 and 10 years (AOR 2.09, 95%CI (1.08, 4.04) and poor quality of life (AOR 3.13, 95%CI (1.79, 5.76) were found to be the factors associated with depression among people with schizophrenia.

**Conclusion:**

The current study revealed that comorbid depression was high among people with schizophrenia and associated with current substance use, suicide attempt, and long duration of the illness as well as poor quality of life. Attention needs to be given to address comorbid depression among people with schizophrenia.

## Background

Globally, depression is one of the major cause of ill health and disability and it is considered as the leading cause of global burden of disease [1, 2]. According to the recent report by WHO the global burden of depression increased by 18% between 2005 to 2015 [2]. Schizophrenia is the most disabling and severe mental disorders with general population lifetime mean prevalence of 0. 4% [3, 4].

Epidemiologic evidence showed that the prevalence of depression is high among people with schizophrenia as compared with the general population [5, 6]. The reported prevalence estimates of depression and depressive symptoms among people with schizophrenia range between 7 to 65% depending on the studies [5, 7–10]. The tools used to measure depression, the severity, duration as well as the various in presenting symptoms of patients with schizophrenia are among the possible reasons for the observed wide range difference in the magnitude of depression in people with schizophrenia.

According to the reports from several studies the presence of comorbid depression in patients with schizophrenia is associated with poor quality of life [11, 12], increased risk of suicide [13, 14], poor treatment adherence [15], frequent relapse and hospitalization [16], disability [17], poor physical health [18], worsening of psychosis [8], as well as poor treatment outcomes [17, 19] as compared to those schizophrenic patients without depression.

Even though depression is considerably common in patients with schizophrenia coupled with its negative consequences, to our knowledge there is no study in Ethiopia that determined the prevalence of depression in patients with schizophrenia. Therefore, this is the first study aimed to explore the prevalence and associated factors of depression in patients with schizophrenia in Ethiopia.

## Methods

### Study setting, design, and period

Institutional based cross-sectional study was performed from October 2017 to March 2018 at Amanuel Mental Specialized Hospital located in the country’s capital, Addis Ababa, Ethiopia. Amanuel mental specialized hospital is the only mental specialized hospital in Ethiopia.

### Study population

The study population consisted of all schizophrenic patients who were on follow up at Amanuel mental specialized hospital during the study period and whose age is 18 years and above on the date of data collection. Those schizophrenic patients who had emergency conditions like aggression (especially physical aggression) were excluded from the current study.

### Sampling procedure and sample size determination

For the current study, we determined sample size based on a single population proportion formula using Epi-info version 7 with a 95% CI, 5% margin of error and taking the prevalence of depression 50% as there were no previous similar studies. By considering a 10% non-response rate a total sample size of 423 schizophrenic patients was included in the study.

We utilized a systematic random sampling technique to select four hundred twenty-three (423) schizophrenic patients who were included in the study. We determined the sampling interval by dividing the total study population (schizophrenic patients) who had follow-up during the study period by the total sample size which was 11(4885/423 = 11) The first study participant was selected using lottery method and the next study participants were choose at a regular interval (every eleventh interval) and interviewed by data collectors.

### Data collection procedures and instrument

Data were collected using pretested an interviewer-administered questionnaire, which contains data on the outcome of interest (depression), socio-demographic characteristics (age, sex, educational status, place of residence, occupation, income, and marital status), clinical factors (duration of the illness, duration of treatment, history of relapse and hospital admission,), quality of life and medication-related factors (quality of life, type of medication, and adherence to the antipsychotics), as well as substance-related factors (khat, alcohol, tobacco and others).

To ascertain the presence of schizophrenia in our study participants, we used a gold standard instrument (Structured Clinical Interview for DSM- ΙѴ-TR axis Ι disorders (SCID). SCID is a diagnostic instrument used to assess DSM-IV-axis I disorders (major mental disorders) and it is extensively used in Ethiopia to assess psychiatric disorders in previous studies [20, 21].

Data on the magnitude of depression was assessed using Patient Health Questionaire-9 (PHQ-9). PHQ-9 was validated in Ethiopia at major referral hospitals with a sensitivity of 86% and specificity of 67%. It categorizes the score as presence and absence of clinically significant depression using a score of 10 as a cutoff point. It also measures severity of the symptoms with cut-off point for normal 0–4, mild depressive symptoms 5–9, Moderate depression 10–14, moderately severe depression 15–19 and for severe depression 20 and above score [22].

To determine the antipsychotic medication adherence, we used Morisky Medication Adherence Scale [23] and we used the World Health Organization Quality of Life-BREF (WHOQOL-BREF) questionnaire to measure the quality of life the study participants [24].

### Data quality control issues

In this study, to control the quality of the study, training was given to the data collectors and supervisors (masters level mental health professionals) on the data collection tool and sampling techniques by the principal investigator. Supervision was conducted regularly during the data collection period both by the researcher, co-investigators and supervisors to check on a daily basis for completeness and consistency. Additionally, a pre-test was conducted 2 weeks before the start of the actual data collection.

### Data processing and analysis

We performed the initial data entry in Epi data and exported to Statistical Package for Social Sciences version 20 (SPSS-20) for further analysis. Descriptive statistics were conducted to quantify the frequencies, mean, standard deviation, and percentages of the variables. Bivariate and multivariate logistic regression was used to see the association between outcome and explanatory variables. The strength of the association was measured by OR with 95% CI and *P*-value less than 0.05 was considered as statistically significant.

### Ethical consideration

Ethical clearance was obtained from Amanuel Mental Specialized Hospital ethical review committee. The four-question version of the abbreviated mental test (AMT4) [25] was used to measure the capacity of the patient to give consent. Then the purpose, objectives, significance, and confidentiality of the collected information were explained to each of the competent study participants before the beginning of the interview. We also informed the participants that there is no harm to them if they would not agree to participate or withdraw from participation during the data collection process. Finally, the study participants willingness to involve in the study was requested and written consent was obtained. During the period of the data collection, the investigator, supervisor and data collectors followed ‘code of ethics’ and obeyed the rules & regulations of the hospital. Privacy was kept confidential at the time of data collection.. All participants who were identified as having depression were referred to psychiatrists for further evaluation and possible treatment with Anti-Depressants.

## Result

### Sociodemographic and medication-related characteristics

Table 1 illustrates the sociodemographic and medication-related characteristics of the participant. In the current study, a total of 418 participants were involved yielding a response rate of 98.8%. The majority of the participants were male (68.7%). The mean age of the participants was 35.50 (standard deviation + 9.25). Majority of the participants (83.6%) were from the urban area. The most frequently prescribed antipsychotic drug was chlorpromazine 180(43%) followed by risperidone 109 (26%). (Table 1).

**Table 1:** The distribution of participants by their socio-demographic and medication related factors, *n* = 422

No.	Variables	Variables category	Frequency	Percentage (100%)
1	Age	18–24	40	9.5
		25–34	167	40
		35–44	145	34.36
		> = 45	66	16.14
2	Sex	Female	130	31.3
		Male	288	68.7
3	Marital Status	Married	152	36.5
		Single	222	53.1
		Divorced and Widowed	44	10.4
4	Educational Status	No formal education	31	7.3
		Primary school	135	32.5
		High School	162	38.9
		Diploma	46	10.9
		Degree and above	44	10.4
5	Occupation	Private	133	32
		Governmental	42	11.1
		Unemployed	140	33.6
		Others**	98	23.2
6	Residence	Urban	351	83.6
		Rural	67	16.4
1	Chlorpromazine	No	238	57
		Yes	180	43
2	Haloperidol	No	357	85.5
		Yes	61	14.5
3	Trifluoperazine	No	413	98.8
		Yes	5	1.2
4	Fluphenazine	No	326	78.2
		Yes	92	21.8
5	Resperidone	No	309	74
		Yes	109	26
6	Olanzapine	No	411	98.3
		Yes	7	1.7
7	Thioridazine	No	398	95.3
		Yes	20	4.7

** House wife, Daily labourers

### Substance-related and clinical characteristics of the participant

In the present study, most of the study participants were living with their illness for more than 5 years (58.8%) and about half of the study participants were on antipsychotic medication for more than 5 years (50.90%). About 26% of the participants were substance users and among the substance users, most of them used cigarette and khat; with 65% each. (Table 2).

**Table 2:** Distribution of participants by clinical and substance use related factors

No	Variable	Variables Category	Frequency	Percentage (100%)
1	Duration of the illness	<=5 years	172	41.2
		6-10 years	117	28.2
		> = 11 years	129	30.6
2	Duration on treatment	<=5 years	205	49.1
		6-10 years	101	24.4
		> = 11 years	112	26.5
3	Admission	No	252	60.2
		Yes	166	39.8
4	Number of admissions	<=1	96	57.1
		> = 2	72	42.9
5	Relapse	No	224	53.6
		Yes	194	46.4
6	Number of relapse	<=1	96	49
		> = 2	100	51
7	Adherence	No	201	48.1
		Yes	217	51.9
8	Current Substance use	No	311	74.2
		Yes	107	25.8
9	Cigarette	No	36	34.9
		Yes	71	65.1
10	Alcohol	No	87	81.7
		Yes	20	18.3
11	Chat	No	36	34.9
		Yes	71	65.1
12	Suicidal Ideation	No	371	88.4
		Yes	47	11.6
13	Suicidal Attempt	No	404	96.2
		Yes	14	3.8

### The prevalence of depression among schizophrenic patients

In this study, the prevalence of depression was 18% with a 95% CI (14.5–22.3). From the total participants with depression, 47.78% had mild, 34.62% had moderate, 15.4% had moderately severe, and 2.2% had severe depression**.**

### Factors associated with depression among schizophrenic patients

In the present study, substance use, duration of illness between 6 and 10 years, suicidality and poor quality of life were found to be a significant associated factors of depression among schizophrenic patients. The multivariable logistic regression revealed that the odds of depression was higher among schizophrenic patients who were substance users (AOR 2.28, 95%CI (1.27, 4.09), suicide attempt (AOR 5.24, 95%CI (2.56, 10.72), duration of illness between 6 and 10 years (AOR 2.09, 95%CI (1.08, 4.04) and poor quality of life (AOR 3.13, 95%CI (1.79, 5.76). (Table 3).

**Table 3:** Factors associated with depression among schizophrenic patients at Amanuel Mental Specialized Hospital Addis, Ababa, Ethiopia, *n* = 418

Explanatory variables	Variables category	Depressive Disorder		Bivariate and Multivariate Analysis		*P*-Value
		Present	Absent	Bivariate Analysis	Multivariate Analysis	
				COR (95% CI)	AOR (95% CI)	
Marital Status	Married	22	128	1.00	1.00	
	Single	37	187	1.07(0.61,1.87)	0.95(0.51,1.78)	
	Divorced	15	29	2.80(1.31,5.99)	1.88(0.80,4.44)	
Current Substance Use	Yes	30	79	1.30(3.72)	2.28(1.27,4.09)	0.005
	No	46	263	1.00	1.00	
Suicide attempt	Yes	23	26	5.34(2.84,10.05)	5.24(2.56,10.72)	0.000
	No	53	316	1.00	1.00	
Adherence	Good Adherence	28	173	1.00	1.00	
	Poor Adherence	48	169	1.75(1.05,2.93)	1.57(0.89,2.77)	
Duration of illness	<=5 Years	25	145	1.00	1.00	
	6 Years–10 Years	30	89	2.01 (1.11,3.63)	2.09(1.08,4.04)	0.028
	> 11 Years	21	108	1.16(0.62,2.18)	0.97(0.48,1.95)	
Quality of life	Good Qol	19	194	1.00	1.00	
	Poor Qol	57	148	4.01(2.29,7.04)	3.13(1.70,5.76)	0.000

## Discussion

### Main findings

To the best of our knowledge, this is the first study that determined the prevalence and associated factors of depression among patients with schizophrenia in Ethiopia. The findings from the current study revealed that a considerable proportion of schizophrenic patients had comorbid depression. The prevalence of depression was found to be 18% (14.5–22.3%) among schizophrenic patients. The prevalence of depression in the current study was in line with the reported magnitude in the USA which was 20% [26]. However, the magnitude of depression in the current study is lower than the reported magnitude in the USA 50% [27], China 54.6% [5], and in Poland 61% [8]. Contrarily the current prevalence is lower than the estimated magnitude of depression in Canada which was 7.3% [9]. Several factors contributed to these wide number of variations in the magnitude of depression among schizophrenic patients across the studies, including: (1) the variations in the instruments used measure depression with different psychometric properties across the studies (i.e. some studies used diagnostic instruments and some used screening instruments); (2) The use of different criteria to categorize depression; (3) the severity of schizophrenic patients; (4) and the presence of other comorbid substance use and medical conditions.

Regarding the associated factors, the current study revealed that factors associated with depression in schizophrenia are substance use, suicidality, longer duration of the illness and poor quality of life. The odds of developing depression were 2.28 times higher in those schizophrenic patients who currently use substance than current non-users (AOR = 2.28, 95%CI (1.27, 4.09)). This is explained by the fact that the direct physiological effect of substance use may result in depressive symptoms. In other words, current substance use in psychiatric patients may double the chance of developing depression.

The patients with a history of suicide attempt were 5.24 times more likely to develop depression compared to those who had no history of suicide attempt (AOR = 5.24, 955CI (2.56, 10.72**)**). This result is in agreement with the findings of the studies performed in Chicago and Poland [8, 28]. This is explained by the reality that suicide attempt goes up to 50% in patients with Schizophrenia and also suicide is one of the symptoms of depression; the presence of depression may also potentiate suicide attempt in this particular population.

Furthermore, in the present study, the patients who lived with the illness for 6–10 years are 2 times more likely to develop depression compared to those who lived with the illness for less than 5 years (AOR = 2.09, 95%CI (1.08,4.04)). This finding is supported by the findings from the study conducted in Poland [8]. It is obvious that throughout the course of Schizophrenia there are multiple episodes of depressive symptoms which range from minimal to severe in intensity. So, the longer the duration of illness the higher the chance of developing Depression.

Finally, patients with poor quality of life were 3.13 times more likely to be depressed compared to those who have a good quality of life (AOR = 3.13 95%CI (1.70,5.76**)**). This finding in agreement with the reported results by James R. Sands and Martin Harrow [29]. Different scholars reported that there is a negative relationship between quality of life and depression; and also, the negative relationship between quality of life and Schizophrenia [29]. This implies that co-presence of depression and Schizophrenia synergistically affect the quality of life of this particular patient.

### The strength and limitation of the study

The current survey had several strengths: (1) the study utilized adequate sample size and the populations were included from a distinct catchment area; (2) our outcome of interest (depression) depression was measured using standard and validated instrument (PHQ-9).

Nevertheless, our study also had some limitations: first, the relationship between the different factors and depression may not imply due to the cross-sectional nature of the study; second, the likelihood of recall bias due to the retrospective nature of cross-sectional studies: thirdly, the findings may not differentiate whether the depressive symptoms are associated with the negative symptoms of schizophrenia or they are purely symptoms of depression because of the cross-sectional nature of the study: fourthly, we didn’t assess substance use disorders.

## Conclusion

The present study revealed that the prevalence of depression among schizophrenic patients was high (18%). There was strong evidence of a positive association between depression and history of substance use, history of suicidality, duration of illness and quality of life among schizophrenic patients. Attention needs to be given to screen and management of depression among schizophrenic patients to alleviate the suffering and prevent further consequences.

## Data Availability

The datasets used and/or analyzed during the current study are available from the corresponding author on reasonable request.

## Abbreviations

DSM: Diagnostic and Statistical Manual of Mental Disorders
PHQ: Patient Health Questionnaire
SCID: Structured Clinical Interview for DSM- ΙѴ-TR axis Ι disorders
WHOQOL: World Health Organization Quality of Life

## References

[1] Whiteford HA, Degenhardt L, Rehm J, Baxter AJ, Ferrari AJ, Erskine HE (2013). Global burden of disease attributable to mental and substance use disorders: findings from the global burden of disease study 2010. Lancet.

[2] WHO (2017). WHO depression fact sheet document 2017.

[3] Goldner EM, Hsu L, Waraich P, Somers JM (2002). Prevalence and incidence studies of schizophrenic disorders: a systematic review of the literature. Can J Psychiatry.

[4] Saha S, Chant D, Welham J, McGrath J (2005). A systematic review of the prevalence of schizophrenia. PLoS Med.

[5] Dai J, Du X, Yin G, Zhang Y, Xia H, Li X (2018). Prevalence, demographic and clinical features of comorbid depressive symptoms in drug naive patients with schizophrenia presenting with first episode psychosis. Schizophr Res.

[6] Rahim T, Rashid R (2017). Comparison of depression symptoms between primary depression and secondary-to-schizophrenia depression. Int J Psychiatry Clin Pract.

[7] Hou C-L, Ma X-R, Cai M-Y, Li Y, Zang Y, Jia F-J (2016). Comorbid moderate–severe depressive symptoms and their association with quality of life in Chinese patients with schizophrenia treated in primary care. Community Ment Health J.

[8] Gozdzik-Zelazny A, Borecki L, Pokorski M (2011). Depressive symptoms in schizophrenic patients. Eur J Med Res.

[9] Bland RC, Newman SC, Orn H (1987). Schizophrenia: lifetime co-morbidity in a community sample. Acta Psychiatr Scand.

[10] Buckley PF, Miller BJ, Lehrer DS, Castle DJ (2008). Psychiatric comorbidities and schizophrenia. Schizophr Bull.

[11] Abedi Shargh N, Rostami B, Kosari B, Toosi Z, Majelan GA (2015). Study of relationship between depression and quality of life in patients with chronic schizophrenia. Global J Health Sci.

[12] Bobes J, Garcia-Portilla MP, Bascaran MT, Saiz PA, Bousoño M (2007). Quality of life in schizophrenic patients. Dialogues Clin Neurosci.

[13] Brugnoli R, Novick D, Haro JM, Rossi A, Bortolomasi M, Frediani S (2012). Risk factors for suicide behaviors in the observational schizophrenia outpatient health outcomes (SOHO) study. BMC Psychiat.

[14] Duko B, Ayano G (2018). Suicidal ideation and attempts among people with severe mental disorder, Addis Ababa, Ethiopia, comparative cross-sectional study. Ann Gen Psychiat.

[15] Higashi K, Medic G, Littlewood KJ, Diez T, Granström O, De Hert M (2013). Medication adherence in schizophrenia: factors influencing adherence and consequences of nonadherence, a systematic literature review. Therap Adv Psychopharmacology.

[16] Ayano G, Duko B (2017). Relapse and hospitalization in patients with schizophrenia and bipolar disorder at the St Amanuel mental specialized hospital, Addis Ababa, Ethiopia: a comparative quantitative cross-sectional study. Neuropsychiatr Dis Treat.

[17] Akinsulore A, Mapayi BM, Aloba OO, Oloniniyi I, Fatoye FO, Makanjuola ROA (2015). Disability assessment as an outcome measure: a comparative study of Nigerian outpatients with schizophrenia and healthy control. Ann General Psychiatry.

[18] Leucht S, Burkard T, Henderson J, Maj M, Sartorius N (2007). Physical illness and schizophrenia: a review of the literature. Acta Psychiatr Scand.

[19] Upthegrove R, Marwaha S, Birchwood M (2017). Depression and schizophrenia: cause, consequence, or trans-diagnostic issue?. Schizophr Bull.

[20] Ayano G, Assefa D, Haile K, Chaka A, Solomon H, Hagos P (2017). Mental, neurologic, and substance use (MNS) disorders among street homeless people in Ethiopia. Ann General Psychiatry.

[21] Duko B, Ayano G, Bekana L, Assefa D (2015). Prevalence and correlates of co-occurring substance use disorder among patients with severe mental disorder at Amanuel mental specialized hospital, Addis Ababa, Ethiopia. J Neuropsychopharmacol Mental Health.

[22] Hanlon C, Medhin G, Selamu M, Breuer E, Worku B, Hailemariam M (2015). Validity of brief screening questionnaires to detect depression in primary care in Ethiopia. J Affect Disord.

[23] Plakas S, Mastrogiannis D, Mantzorou M, Adamakidou T, Fouka G, Bouziou A (2016). Validation of the 8-item Morisky medication adherence scale in chronically ill ambulatory patients in rural Greece. Open J Nursing.

[24] Group W (1998). Development of the World Health Organization WHOQOL-BREF quality of life assessment. Psychol Med.

[25] Woodford HJ, George J (2007). Cognitive assessment in the elderly: a review of clinical methods. QJM.

[26] Zisook S, McAdams LA, Kuck J, Harris MJ, Bailey A, Patterson TL (1999). Depressive symptoms in schizophrenia. Am J Psychiatr.

[27] Chemerinski E, Bowie C, Anderson H, Harvey PD (2008). Depression in schizophrenia: methodological artifact or distinct feature of the illness?. J Neuropsychiat Clin Neurosciences.

[28] Sands JR, Harrow M (1999). Depression during the longitudinal course of schizophrenia. Schizophr Bull.

[29] Harrow M, Yonan CA, Sands JR, Marengo J (1994). Depression in schizophrenia: are neuroleptics, akinesia, or anhedonia involved?. Schizophr Bull.

